# Associations between basic motor competencies and physical fitness in Spanish pre-schoolers: a cross-sectional study

**DOI:** 10.1186/s13052-023-01504-w

**Published:** 2023-08-11

**Authors:** Gaizka Legarra-Gorgoñon, Yesenia García-Alonso, Robinson Ramírez-Vélez, Blanca Erice-Echegaray, Mikel Izquierdo, Alicia M. Alonso-Martínez

**Affiliations:** 1 Department of Health Sciences, Navarrabiomed, Hospital Universitario de Navarra (HUN), Universidad Pública de Navarra (UPNA), Instituto de Investigación Sanitaria de Navarra (IdiSNA), Avenida de Barañain S/N, Navarra 31008 Pamplona, Spain; 2 CIBER of Frailty and Healthy Aging (CIBERFES), Instituto de Salud Carlos III, Madrid, Spain

**Keywords:** Motor skills, Physical fitness, Pre-schoolers, Children

## Abstract

**Background:**

Basic motor competencies (BMC) play a vital role in child development and are a primary focus of physical education. However, there is a lack of research on BMC in preschool-aged children, making this study particularly significant. The present study aims to examine the relationship between BMC and physical fitness components in Spanish preschool children.

**Methods:**

This cross-sectional study was conducted on 101 children (*n* = 52 boys, average age of 4.80 years) living in Pamplona, North Spain. Components of physical fitness (handgrip strength, standing long jump, speed-agility, and cardiorespiratory fitness) were assessed using the PREFIT Battery. Overall physical fitness was computed from the four selected fitness components separately for boys and girls. Higher z-scores values in physical fitness indicate better fitness performance. To assess BMC, the MOBAK KG test battery was applied (subscales of object control and self-movement). Sex, age, and body mass index were used as main confounders.

**Results:**

After adjustment for confounding factors, the BMC was positively associated with single (z-scores) components and overall physical fitness sum of z-scores (*R*^2^ from 0.178 to 0.469, all *P*-values < 0.05). Additional percentile scores weakened the associations, but they still remained significant (*R*^2^ from 0.146 to 0.337, all *P*-values < 0.05). Compared with participants in the lowest tertile, those in the highest tertile of the MOBAK KG sum score, and in both object movement and self-movement test subscales had significantly higher scores in single fitness component (z-scores or percentile) and overall physical fitness sum of z-scores (all *P* for trend < 0.001).

**Conclusions:**

Our results revealed that higher BMC is associated with better physical fitness in Spanish preschool children. The findings emphasize the crucial role that basic motor competence plays in promoting physical activity in preschool children.

**Supplementary Information:**

The online version contains supplementary material available at 10.1186/s13052-023-01504-w.

## Introduction

Physical inactivity among children and adolescents is and emerging public health problem [1]. In 2018, only between 20 and 40% of 5- to 17-year-old children in Europe met the WHO recommendations of 60 min moderate to vigorous intensity physical activity daily [2]. In view of these negative trends and the importance of physical activity in health, research has focused on understanding the underlying mechanisms of factors in order to promote an active and healthy lifestyle. In 2008, Stodden et al. [3] developed a model that discussed the role of motor competence and physical fitness in physical activity. Their authors hypothesized that the relationship between basic motor competence (BMC) and physical fitness is reciprocal and changes over time.

A key factor that was described in the model of Stodden et al. [3] is physical fitness. Physical fitness is a crucial indicator of health in children, involving physical and physiological components such as cardiorespiratory fitness, musculoskeletal fitness (i.e., muscular endurance and strength), and flexibility [4]. Research has indicated negative correlations between changes in cardiorespiratory fitness (CRF) and obesity later in life [5, 6], as well as being a significant predictor of morbidity and mortality in adults independent for smoking status and alcohol [7].

In their model, Stodden et al. [3] postulated that physical fitness components are positively associated with BMC and/or physical activity levels. In this line, previous studies have consistently shown that BMC is directly associated with several physical fitness subdomains among children and adolescents [8, 9]. Recently, Utesch et al. [10] conducted a systematic review on the associations between motor competence and subdomains of physical fitness (i.e., CRF and musculoskeletal fitness) in early childhood. The authors revealed moderate to large positive associations between motor competence and physical fitness, findings which are consistent with previous literature reviews [8, 9, 11].

BMC play a vital role in child development and are a primary focus of physical education. Children aged 3–6 years are at a crucial stage in improving their motor skills, as everything they do involves motor actions such as posture, movement, exploration, social interaction, and object manipulation [12]. However, there is a lack of research on BMC in preschool-aged children, making this study particularly significant [13]. A validated and simple tool for assessing BMC is the MOBAK KG test, which evaluates two competence areas: object movement and self-movement [14].

There is a need to further explore the available evidence on the association between BMC and physical and physiological components of physical fitness. Therefore, the objective of this study is to examine the relationship between BMC (assessed by MOBAK KG and MOBAK total score) and components of physical fitness (handgrip strength, standing long jump, speed/agility, and cardiorespiratory fitness) in Spanish children aged 4–5 years using the PREFIT battery. Based on previous research and theoretical considerations, it is hypothesized that physical fitness is associated with BMC. With the growing concern of childhood obesity and physical inactivity, this research could provide valuable insight into the impact of BMC on physical fitness and overall health in early childhood.

## Methods

### Study design and participants

The present study used cross-sectional data obtained from baseline assessments of “*in spanish* observatorio y programa de intervención de ejercicio físico y estilos de vida en familia para niños y niñas de 4 a 6” (https://observatorioactividadfisica.es). A sample of 101 children (52 boys and 49 girls) with an average age of 4.80 years was analyzed. Between February and March 2022, the parents of the children were informed of the study's objectives and given the opportunity to review the study protocol. Participants were recruited from a pool of 4-year-old children registered at the Iturrama medical center (Pamplona, Spain). Exclusions included children with recent injury/surgery or medical limitations on physical testing. Oral assent and written informed consent were provided by the children and their legal guardians, respectively. All procedures were in line with the Declaration of Helsinki and ethical approval was granted by the local ethics committees at Universidad Pública de Navarra (PI_2021/111).

### Measures and procedures

All participants’ body height and weight was measured with a stadiometer and an electronic scale following the CDC-NHANES Survey protocol by trained evaluators [11]. Height was measured in the Frankfurt position with a SECA 213® stadiometer (1-mm precision), and weight was measured using a Tanita DC-430MAS® scale (100-g precision), while participants were in light clothing and bare feet. BMI was calculated as kg/m^2^ using measured weight and height. Waist circumference was measured to the nearest 1 mm by using a calibrated anthropometric tape measure at the umbilicus.

Physical fitness (CRF, lower- and upper-body muscular strength and speed-agility) was measured using the PREFIT test battery [15]. The tests were performed in a circuit by the same researchers with a previous demonstration of the test. Participants performed each test consecutively, except for the 20-m shuttle run, where several children ran at the same time. Upper-body muscular strength was measured using a handgrip strength (HGS). Children squeezed an analog dynamometer (TKK 5001, Grip-A, Takei, Tokyo) gradually and continuously for at least 2 to 3 s. The test was performed twice for each hand. The highest value for each hand was retained and then the average for these two results was used in the analyses as a measure of upper-body muscular strength. Lower-body muscular strength was assessed using the standing long jump test (SLJ). Briefly, children performed a maximal horizontal jump from a stationary standing position, landing on both feet while maintaining an upright posture. The better of three attempts was used in the analyses. For the speed/velocity, the children ran as fast as possible between two lines that were 10 m apart covering a total of 40 m. In this test, lower scores indicate higher performance. This test was performed twice, and the quickest time was used in the analyses. To conduct CRF testing, children were required to run between two lines that were 20 m apart. An audio signal was used, which gradually accelerated starting at a speed of 6.5 km/h and increased by 0.5 km/h per minute. The test would conclude when the child failed to reach one of the lines for two consecutive times with the audio signal, or when the child finished due to exhaustion. Each child performed the test once, and the results were expressed as the number of laps completed. The percentile for each physical fitness component was determined for each child using the PREFIT percentile calculator [15].

In the end, the standardized values (z-score) of each test were calculated using the mean value of each test minus each students’ value, then the difference value was divided by the standard deviation of each test. A continuous score was computed from the four selected fitness component separately for boys and girls. Higher z-scores values in physical fitness indicate better fitness performance.

The MOBAK KG test battery was used to assess BMC [14]. This battery includes a total of eight test items and is designed to measure the basic motor competencies of self-movement (items: balancing, rolling, jumping, running) and object movement (items: throwing, catching, bouncing, dribbling) in preschoolers aged between four and six years. Accordingly, in both MOBAK KG test subscales (self-movement and object movement) maximum scores of 8 points can be reached, resulting in a combined MOBAK KG sum score ranging from 0 (lowest BMC) to 16 points (highest BMC).

Before performing the tasks, participants were not given any prior attempts to practice. For the “throwing” and “catching” tasks, participants had six attempts to complete the task. Scoring was based on the number of successful attempts, with 0–2 attempts scoring 0 points, 3–4 attempts scoring 1 point, and 5–6 attempts scoring 2 points. In the “bouncing”, “dribbling”, “balancing”, “rolling”, “jumping”, and “running” tasks, participants had two attempts to complete each task. These items were scored dichotomously (0 = failed; 1 = passed), with the number of successful attempts recorded. A score of 0 points was awarded for 0 successful attempts, 1 point for 1 successful attempt, and 2 points for 2 successful attempts. The MOBAK test items provide detailed information regarding which test item each child passed, as well as identifying areas where additional support is needed. Additionally, the MOBAK KG scores were categorized into tertiles, representing low, medium, and high values. This categorization was performed to examine the associations between physical fitness and BMC.

### Statistical analysis

Analyses were performed using the Statistical Package for the Social Sciences (IBM SPSS Statistics 26 version for Windows; SPSS Inc., Chicago, IL). Continuous variables were expressed as means ± standard deviation (SD). The Kolmogorov–Smirnov tests were used for normality of distribution tests. All categorical variables were presented by frequencies and percentages. The mean difference of continuous variables among sex groups was compared using an independent samples *t* test. To compare proportions between sex, chi-square test was used. Multiple linear regression analysis was performed to establish the relationships between BMC and physical fitness (e.g., standardized values z-score/percentiles for each component and overall fitness Z-score). Potential confounders such as sex, age and BMI were used as covariates in the analyses. The association of standardized values z-score/percentiles for each component and overall fitness Z-score with MOBAK KG sum score, as well as with MOBAK KG test subscales (e.g., self-movement, object movement and sum score) separately, was assessed by linear regression analysis.

Finally, analysis of covariance (ANCOVA) was used to assess the difference in overall physical fitness and their components (e.g., standardized values z-score and percentiles) across different MOBAK KG tertiles groups, adjusted for sex, age and BMI as covariates. Post hoc analyses were conducted with the Bonferroni adjustment. All physical fitness variables with a non-normal distribution were z-scores values transformed for linear regression and ANCOVA analyses. Values of *P* < 0.05 were considered statistically significant.

## Results

Table 1 presents a summary of the descriptive characteristics of the children in the sample, including their anthropometric measures, physical fitness components, and MOBAK KG scores. The sample consisted of 101 children, including 49 girls and 52 boys, with a mean age of 4.80 ± 0.51 years. Boys were taller (*P* = 0.006) and slightly heavier (*P* = 0.071) than girls, although mean BMI was similar between genders (*P* = 0.652). Boys showed higher upper-body muscular fitness and object movement scores than girls (*P* < 0.05).

**Table 1:** Characteristics of the study population

Characteristics	Girls (*n* = 49)	Boys (*n* = 52)	Total (*n* = 101)	*P*-value
**Anthropometric parameters**				
Age	4.72 ± 0.45	4.88 ± 0.56	4.80 ± 0.51	0.123
Height (cm)	105.92 ± 4.72	108.71 ± 5.07	107.36 ± 5.08	0.006
Weight (kg)	18.38 ± 2.79	19.54 ± 3.50	18.98 ± 3.21	0.071
Body mass index (kg/m^2^)	16.30 ± 1.45	16.46 ± 2.10	16.38 ± 1.81	0.652
Waist circumference (cm)	53.90 ± 3.43	54.22 ± 5.80	54.06 ± 4.78	0.735
Maternal education^a^	43	57	49	0.275^c^
Monthly family income^b^	43	56	50	0.540^c^
**Physical fitness components**				
Handgrip strength (kg)	7.09 ± 2.13	8.23 ± 2.11	7.67 ± 2.19	0.009
Standing long jump (cm)	84.47 ± 16.65	87.16 ± 23.37	85.88 ± 20.37	0.530
Speed/agility (s)	15.90 ± 1.71	15.60 ± 1.70	15.75 ± 1.70	0.395
Cardiorespiratory fitness (laps)	23.98 ± 11.39	27.26 ± 12.23	25.64 ± 11.87	0.189
Overall fitness (sum of z-scores)	-0.10 ± 0.71	0.13 ± 0.75	0.01 ± 0.74	0.144
**MOBAK KG**				
Object movement subscale (8 points)	1.80 ± 1.89	2.71 ± 2.33	2.26 ± 2.16	0.035
Self-movement subscale (8 points)	2.68 ± 2.01	3.12 ± 2.5	2.90 ± 2.27	0.336
MOBAK KG sum score	4.48 ± 3.46	5.82 ± 4.26	5.16 ± 3.91	0.085

Data are presented as mean ± standard deviation, except for maternal education and monthly family income as percentage (%)

^a^Mother with university studies

^b^More or equal than 3000 euros (€)

^c^Differences between boys and girls were examined by analysis of the by *t* test, except for maternal education and monthly family income (chi-square test)

**Fig. 1:**
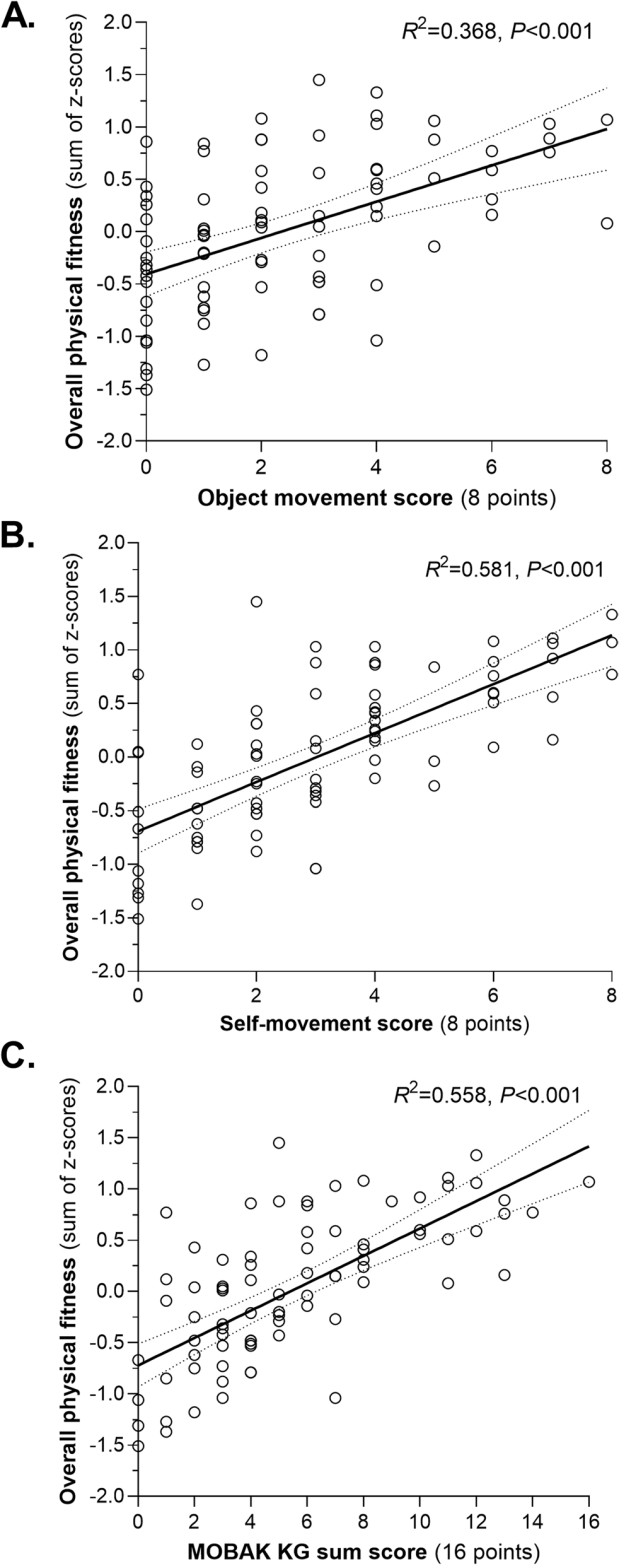
Relationship for MOBAK KG sum score and subscales (object movement and self-movement test) with overall physical fitness (sum of z-score)

Figure 1 shows positive relationship for MOBAK KG and test subscales (object movement and self-movement) with overall physical fitness (sum of z-score). Weak to moderate negative associations were observed for object movement (*R*^2^ = 0.368, *P* < 0.001; Fig. 1A), self-movement (*R*^2^ = 0.581, *P* < 0.001; Fig. 1B) and MOBAK KG sum score (*R*^2^ = 0.558, *P* < 0.001; Fig. 1C) with overall physical fitness (sum of z-score).

Table 2 shows the association between MOBAK KG scores (self-movement, object movement and sum score), physical fitness components (Z-score) and overall physical fitness (sum of z-score). Results indicate that BMC is significantly related to z-score HGS (*R*^2^ from 0.331 to 0.357, all *P*-values < 0.05), z-score SLJ (*R*^2^ from 0.171 to 0.345, all *P*-values < 0.05), z-score speed/agility (*R*^2^ from 0.299 to 0.469, all *P*-values < 0.05), z-score CRF (*R*^2^ from 0.295 to 0.387, all *P*-values < 0.05) and overall physical fitness (sum of z-score) (*R*^2^ from 0.368 to 0.581, all *P*-values < 0.05).

**Table 2:** Unstandardized regression coefficients (β) examining the association of MOBAK KG score and test subscales (object movement and self-movement) for Z-scores and percentiles

Dependent variable	Object movement score (8 points)				Self-movement score (8 points)				MOBAK KG sum score (16 points)			
	β	95% IC	R^2^	*P* value	β	95% IC	R^2^	*P* value	β	95% IC	R^2^	*P* value
**Z-score**												
Handgrip strength (kg)	0.048	-0.033 – 0.128	0.331	0.244	0.105	0.032 – 0.179	0.375	0.006	0.050	0.007 – 0.094	0.357	0.024
Standing long jump (cm)	0.154	0.060 – 0.248	0.178	0.002	0.241	0.160 – 0.322	0.345	< 0.001	0.131	0.083 – 0.180	0.309	< 0.001
Speed/agility (s)	0.216	0.128 – 0.303	0.299	< 0.001	0.279	0.205 – 0.353	0.436	< 0.001	0.166	0.122 – 0.209	0.469	< 0.001
Cardiorespiratory fitness (laps)	0.219	0.132 – 0.305	0.295	< 0.001	0.231	0.152 – 0.310	0.345	< 0.001	0.144	0.100 – 0.189	0.386	< 0.001
Overall fitness (sum of z-scores)	0.155	0.092 – 0.219	0.368	< 0.001	0.217	0.167 – 0.267	0.581	< 0.001	0.123	0.093 – 0.153	0.558	< 0.001
**Percentile score**												
Handgrip strength (kg)	2.571	-0.159 – 5.300	0.087	0.065	4.778	2.390 – 7.167	0.193	< 0.001	2.430	0.992 – 3.867	0.157	0.001
Standing long jump (cm)	1.451	-1.222 – 4.124	0.112	0.283	4.928	2.529 – 7.327	0.253	< 0.001	2.228	0.743 – 3.713	0.189	0.004
Speed/agility (s)	5.044	2.009 – 8.080	0.146	0.001	7.895	5.304 – 10.486	0.337	< 0.001	4.590	3.003 – 6.178	0.315	< 0.001
Cardiorespiratory fitness (laps)	5.749	3.151 – 8.347	0.204	< 0.001	6.585	4.226 – 8.944	0.283	< 0.001	3.971	2.627 – 5.315	0.306	< 0.001

Coefficients are adjusted for sex, age and BMI

Additional percentile scores weakened the associations, but they still remained significant, between BMC and HGS (*R*^2^ from 0.193 to 0.157, all *P*-values < 0.05), SLJ (*R*^2^ from 0.189 to 0.253, all *P*-values < 0.05), speed/agility (*R*^2^ from 0.146 to 0.337, all *P*-values < 0.05), and CRF (*R*^2^ from 0.204 to 0.306, all *P*-values < 0.05).

**Fig. 2:**
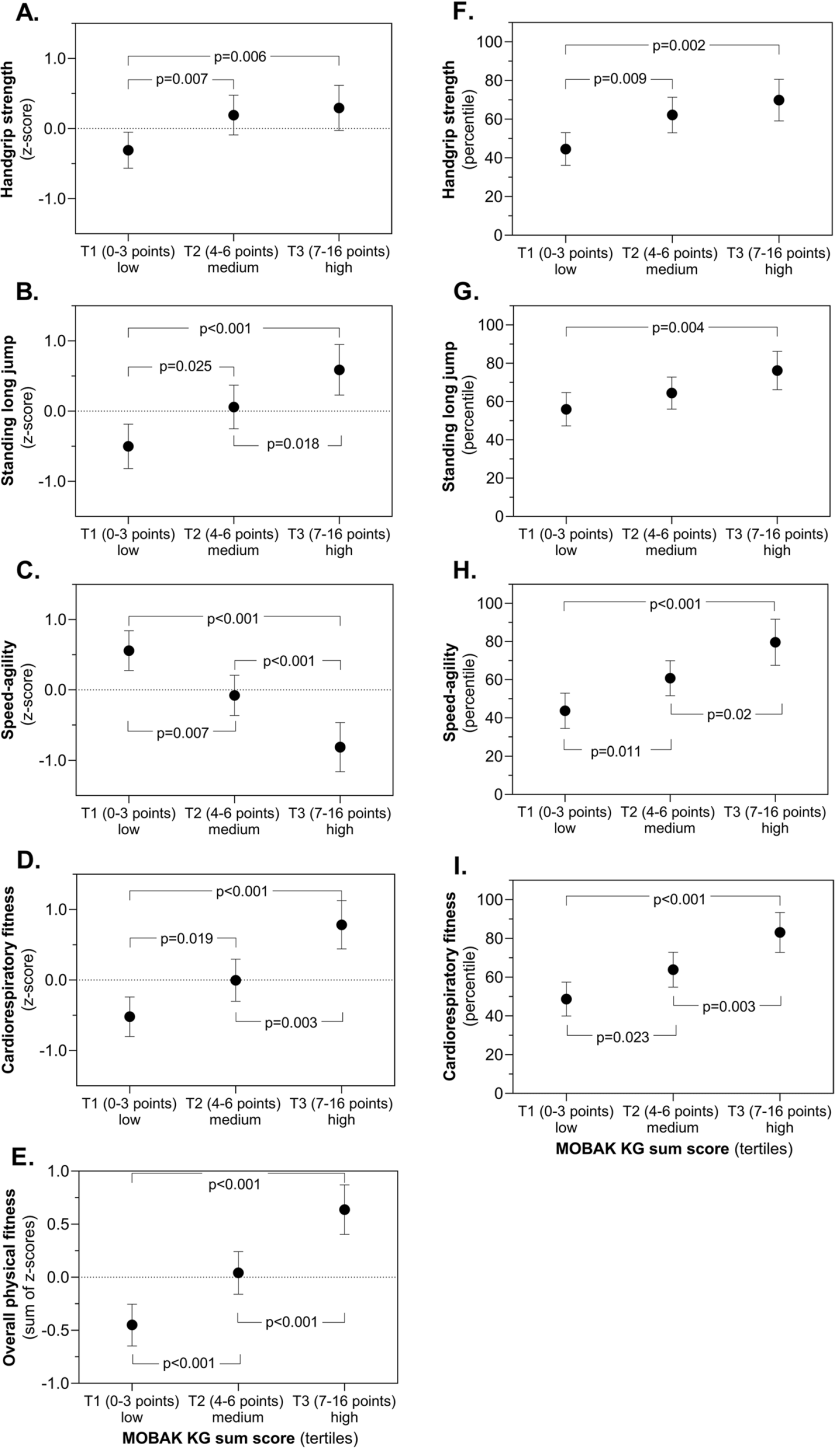
Single (z-scores and percentiles) components and overall physical fitness sum of z-scores by tertiles of MOBAK KG sum score (analysis of covariance, sex, age and body mass index as confounders)

The association between MOBAK KG sum score with a single fitness component (z-scores or percentile) and overall physical fitness (sum of z-scores) are depicted in Fig. 2. In this analysis, participants were categorized based on tertiles (T) of MOBAK KG sum score, and in both object movement and self-movement test subscales. Higher tertiles of MOBAK KG score demonstrate better motor competence performance compared to lower tertiles. Compared with children in the lowest/medium tertile, those in the highest tertile of the MOBAK KG score had significantly higher fitness component Z-scores values for the HGS (T1 vs. T2 *P* = 0.007; T1 vs. T3 *P* = 0.006; Fig. 2A), SLJ (T1 vs. T2 *P* = 0.025; T1 vs. T3 *P* < 0.001; T2 vs. T3 p = 0.018; Fig. 2B), speed/agility (T1 vs. T2 *P* = 0.007; T2 vs. T3 *P* < 0.001; T1 vs. T3 *P* < 0.001; Fig. 2C), CRF (T1 vs. T2 *P* = 0.019; T2 vs. T3 *P* = 0.003; T1 vs. T3 *P* < 0.001; Fig. 2D), and overall physical fitness sum of z-scores (T1 vs. T2 *P* < 0.001; T2 vs. T3 *P* < 0.001; T1 vs. T3 *P* < 0.001; Fig. 2E). These associations were further explored separately in both object movement and self-movement test subscales, and they are presented as online Supporting Information Figure S1.

There were associations that remained significant between BMC and the four selected fitness component percentiles scores: SLJ (T1 vs. T3 *P* = 0.004; Fig. 2F), HGS (T1 vs. T2 *P* = 0.009; T1 vs. T3 *P* = 0.002; Fig. 2G), speed/agility (T1 vs. T2 *P* = 0.011; T2 vs. T3 *P* = 0.02; T1 vs. T3 *P* < 0.001; Fig. 2H), and CRF (T1 vs. T2 p = 0.023, T2 vs. T3 *P* = 0.003; T1 vs. T3 *P* < 0.001; Fig. 2I). These associations were further explored separately in both object movement and self-movement test subscales, and they are presented as online Supporting Information Figure S2.

## Discussion

The present study aimed to examine the connections between BMC (assessed in two subscales using the MOBAK KG test battery), four components of physical fitness (HGS, SLJ, speed/agility, and CRF) and overall physical fitness sum of z-scores. The main finding of this study is that higher MOBAK KG score and test subscales (object movement and self-movement) are associated with a healthier level of physical fitness (i.e., z-score and percentiles) in Spanish pre-schoolers. Moreover, our results confirm that better motor competence performance children (highest tertile) have better single fitness component (z-scores or percentile) and overall physical fitness (sum of z-scores) than their lowest tertile peers. This study holds significance due to the limited amount of research available on the assessment of BMC and the establishment of normative values in pre-school-aged children.

Previous studies have shown that BMC differences between boys and girls increase as they age [4]. The MOBAK test battery has been validated as a reliable tool for evaluating motor performance in primary school children [14]. Previous studies have found that boys typically exhibit higher motor competence in object manipulation tasks, while girls tend to score higher in stability and balance tasks [16]. Our findings align with this pattern, with boys showing significantly higher motor proficiency in object manipulation tasks. However, in self-movement tasks, there was no significant difference between the scores of boys and girls. This may partially be due to the young age of our study participants. Hraski et al. [17] investigated the kinematic parameters and morphological characteristics of boys, revealing a negative correlation between motor performance and indicators of wellbeing. On the other hand, a positive correlation was observed between morphological measures such as body mass, subcutaneous fat tissue and body fat percentage, and explosive strength. Moreover, it was found that motor competences are higher in boys, even during infancy, which may explain their superior performance in fitness tests. Before the onset of adolescence, boys and girls will follow similar rates of maturation even though boys will consistently outperform girls in a range of biomotor skills (e.g., speed, strength, and endurance). The level of motor activity is higher in boys even in infancy, and gender differences increase across childhood and adolescence.

Our results provide evidence supporting previous research that connects motor skills, body mass composition, and physical fitness. Several studies [18, 19] have demonstrated that higher levels of BMC are correlated with better overall fitness in children, and that motor skills are linked to measures of speed, agility, CRF, and handgrip strength. Additionally, it has been shown that higher levels of BMC are positively related to physical activity levels in children and adolescents. In the same context, there is evidence demonstrating the influence of the muscle mass on physical fitness, which could serve as a potential mechanism underlying these relationships. Previous cross-sectional studies [20, 21] have demonstrated that a lower fat max index is associated with higher performance in the physical measures such as 20-m shuttle run, standing long jump and 4x10 m speed/agility test.

The observations of the present study are limited by the cross-sectional design nature, and causality cannot be determined. Prospective and more controlled intervention studies are therefore required to draw more robust conclusions. Also, the non-representative sample size, and the recruitment of participants from only one health center are limitations of the present study. Lastly, normative values for motor skills in Spanish children of this age and using this test battery are currently lacking. Nevertheless, the current study has strengths. The use of BMC values measured using the MOBAK KG battery in Spanish children aged 4–5 years old is an important strength in the present study. Moreover, the reliability and validity of PREFIT-fitness [15] tests for measuring fitness in this population, is a remarkable strength.

## Conclusions

The findings of this study underscore the significant association between BMC and physical fitness, highlighting the importance of promoting motor competence to enhance levels of physical activity. Previous studies examining BMC have consistently reported positive outcomes following the implementation of various programs with differing durations (ranging from 2 to 10 months), frequencies (2–3 days per week), and settings (school or home) [13]. These interventions have successfully improved BMC in preschool-aged children. A wide range of activities, including jumping, ball throwing at a target, walking, and running, among others, have demonstrated significant differences in BMC between pre- and post-training stages [22, 23]. Future health and fitness programs should consider ways to enhance BMC in order to improve the overall fitness and health of children.

### Supplementary Information

**Additional file 1: Figure S1.** Single (z-scores and percentiles) components and overall physical fitness sum of z-scores by tertiles of MOBAK KG (object movement test) subscale (analysis of covariance, sex, age and body mass index as confounders). **Figure S2.** Single (z-scores and percentiles) components and overall physical fitness sum of z-scores by tertiles of MOBAK KG (self-movement test) subscale (analysis of covariance, sex, age and body mass index as confounders).

## Data Availability

Not applicable.

## Abbreviations

BMC: Basic Motor Competences
BMI: Body mass index
CRF: Cardiorespiratory fitness
HGS: Handgrip strength
MOBAK KG: Motorsiche Basiskompetenzen Kindergarden
MTS: Mobak Total Score
OM: Object movement
PF: Physical fitness
SLJ: Standing long jump
SM: Self movement
T: Tertile
