# Supplementary material for: Associations between basic motor competencies and physical fitness in Spanish pre-schoolers: a cross-sectional study
Source: Ital J Pediatr. 2023 Aug 11;49:97. doi: 10.1186/s13052-023-01504-w (PMC10422786; doi:10.1186/s13052-023-01504-w)
Supplement: Supplementary file 1 — Additional file 1: Figure S1. Single (z-scores and percentiles) components and overall physical fitness sum of z-scores by tertiles of MOBAK KG (object movement test) subscale (analysis of covariance, sex, age and body mass index as confounders). Figure S2. Single (z-scores and percentiles) components and overall physical fitness sum of z-scores by tertiles of MOBAK KG (self-movement test) subscale (analysis of covariance, sex, age and body mass index as confounders). [file 13052_2023_1504_MOESM1_ESM.doc]

**Associations Between Basic Motor Competencies and Physical Fitness in Spanish Pre-schoolers:** **a Cross-Sectional Study**

Gaizka Legarra-Gorgoñon^1^, Yesenia García-Alonso^1^, Robinson Ramírez-Vélez^1,2^, Blanca Erice-Echegaray^1^, Mikel Izquierdo^1,2^, Alicia M. Alonso-Martínez^1^

1. Navarrabiomed, Hospital Universitario de Navarra (HUN), Universidad Pública de Navarra (UPNA), Instituto de Investigación Sanitaria de Navarra (IdiSNA), Pamplona, Spain.

2. CIBER of Frailty and Healthy Aging (CIBERFES), Instituto de Salud Carlos III, Madrid, Spain.


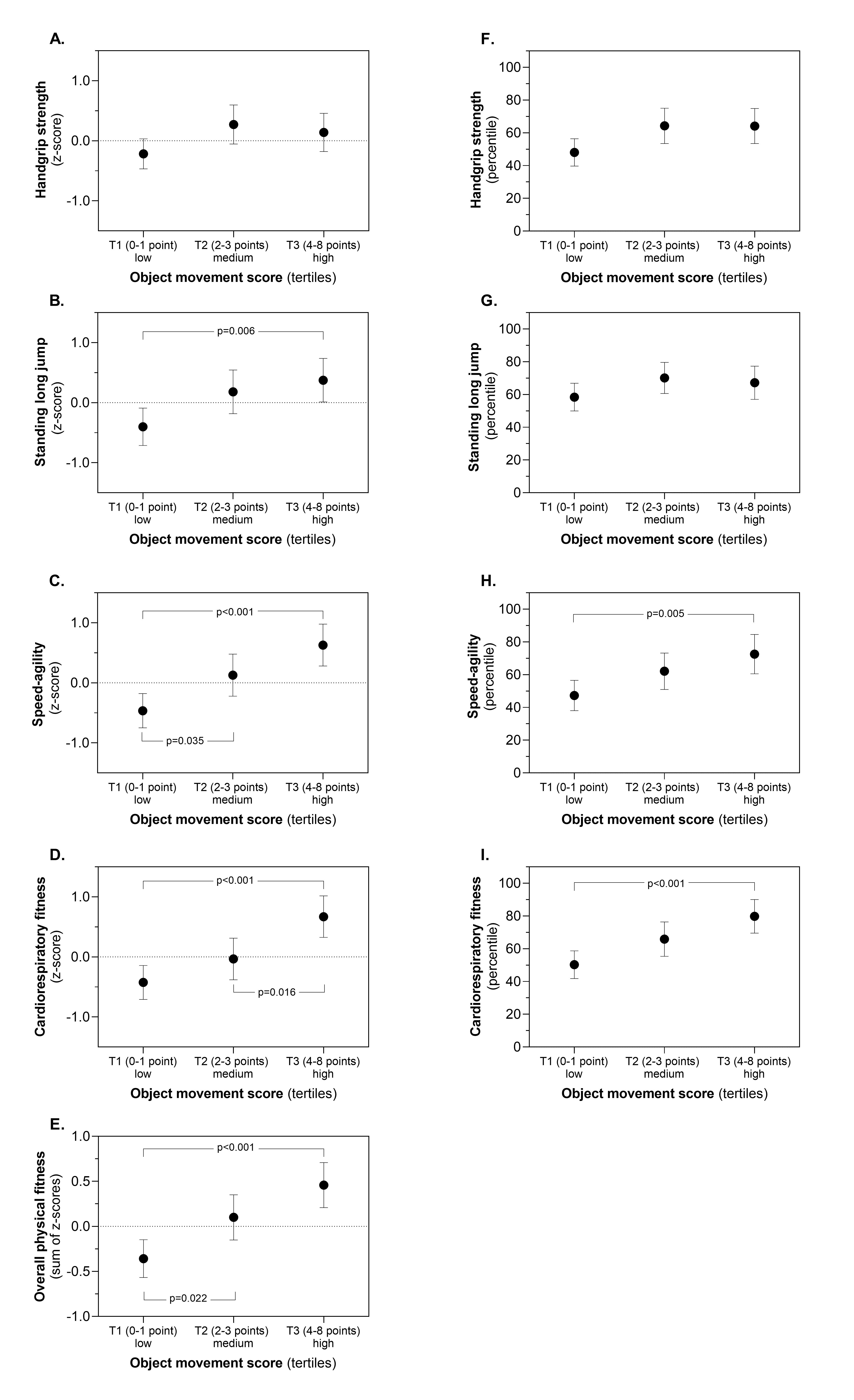


**Figure S1.** Single (z-scores and percentiles) components and overall physical fitness sum of z-scores by tertiles of MOBAK KG (object movement test) subscale (analysis of covariance, sex, age and body mass index as confounders).


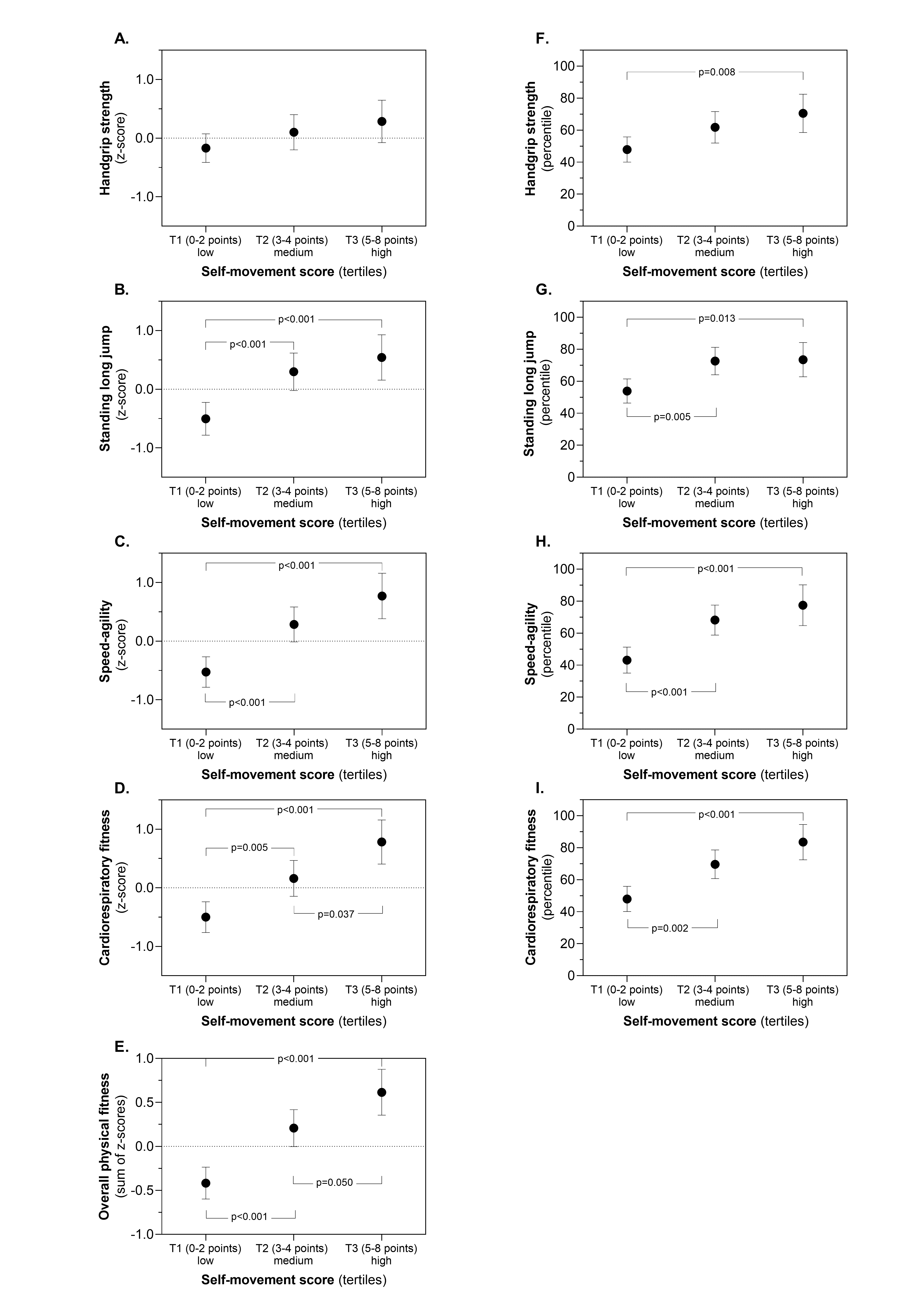


**Figure S2.** Single (z-scores and percentiles) components and overall physical fitness sum of z-scores by tertiles of MOBAK KG (self-movement test) subscale (analysis of covariance, sex, age and body mass index as confounders).
